# Crystal structure, Hirshfeld surface analysis and DFT studies of 2-[4-(2-methyl­prop­yl)phen­yl]-*N*′-[(1*Z*)-1-(thio­phen-2-yl)ethyl­idene]propane­hydrazide

**DOI:** 10.1107/S2056989025003329

**Published:** 2025-04-24

**Authors:** Sarayu Jayadevan, K. V. Sujith, A. R. Biju

**Affiliations:** a Department of Chemistry, Sir Syed College, Taliparamba, Kannur, 670 142, India; b Department of Chemistry Kannur University, Swami Anandatheertha Campus Edat PO Payyanur 670 327 Kerala India; c Department of Chemistry, Payyanur College, Edat PO, Payyanur, 670 327, Kerala, India; Indian Institute of Science Education and Research Bhopal, India

**Keywords:** crystal structure, Hirshfeld surface analysis, single crystal X-ray diffraction, inter­action energy, ibuprofen hydrazide

## Abstract

In the title compound C_19_H_24_N_2_OS, inter­molecular N—H⋯O hydrogen bonds generate *R*_2_^2^(8) ring motifs, forming dimers with an inter­action energy of −70.5 kJ mol^−1^. A short S⋯C inter­action produces another dimer with an inter­action energy of −30.6 kJ mol^−1^. The inter­molecular inter­actions were qu­anti­fied using Hirshfeld surface analysis. The two-dimensional fingerprint plots indicate that the major contributions to the crystal packing are from H⋯H (67.9%), C⋯H (13.7%), O⋯H (7.3%) and S⋯H (4.3%) inter­actions.

## Abstract 2

In the crystal of the title compound, N—H⋯O hydrogen bonds lead to the formation of dimers with an inter­action energy of −70.5 kJ mol^−1^. The two-dimensional fingerprint plots indicate that the major contributions to the crystal packing are from H⋯H (67.9%), C⋯H (13.7%), O⋯H (7.3%) and S⋯H (4.3%) inter­actions.

## 1. Chemical context

Derivatives of ibuprofen have been synthesized to enhance the efficacy and reduce the side effects commonly associated with traditional NSAIDs (Ahmadi *et al.*, 2017 ▸). These deriv­atives have shown promising results in preliminary studies, particularly regarding anti-inflammatory, analgesic, and anti­microbial activities (Sujith *et al.*, 2009 ▸; Dhakane *et al.*, 2014 ▸). Compared to other derivatives of NSAIDs, ibuprofen hydrazides may offer distinct advantages, although comprehensive clinical comparisons are still limited (Kamms & Hadi, 2023 ▸). Based on the above studies, we herein report the crystal structure of the ibuprofen hydrazide derivative 2-[4-(2-methyl­prop­yl)phen­yl]-*N*′-[(1*Z*)-1-(thio­phen-2-yl)ethyl­idene]propane­hydrazide (**1**).
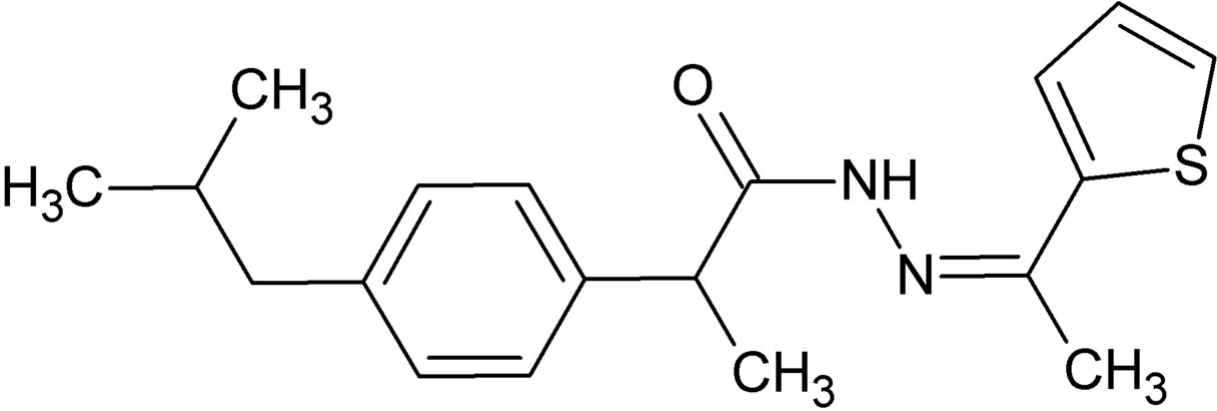


## 2. Structural commentary

**Figure 1:**
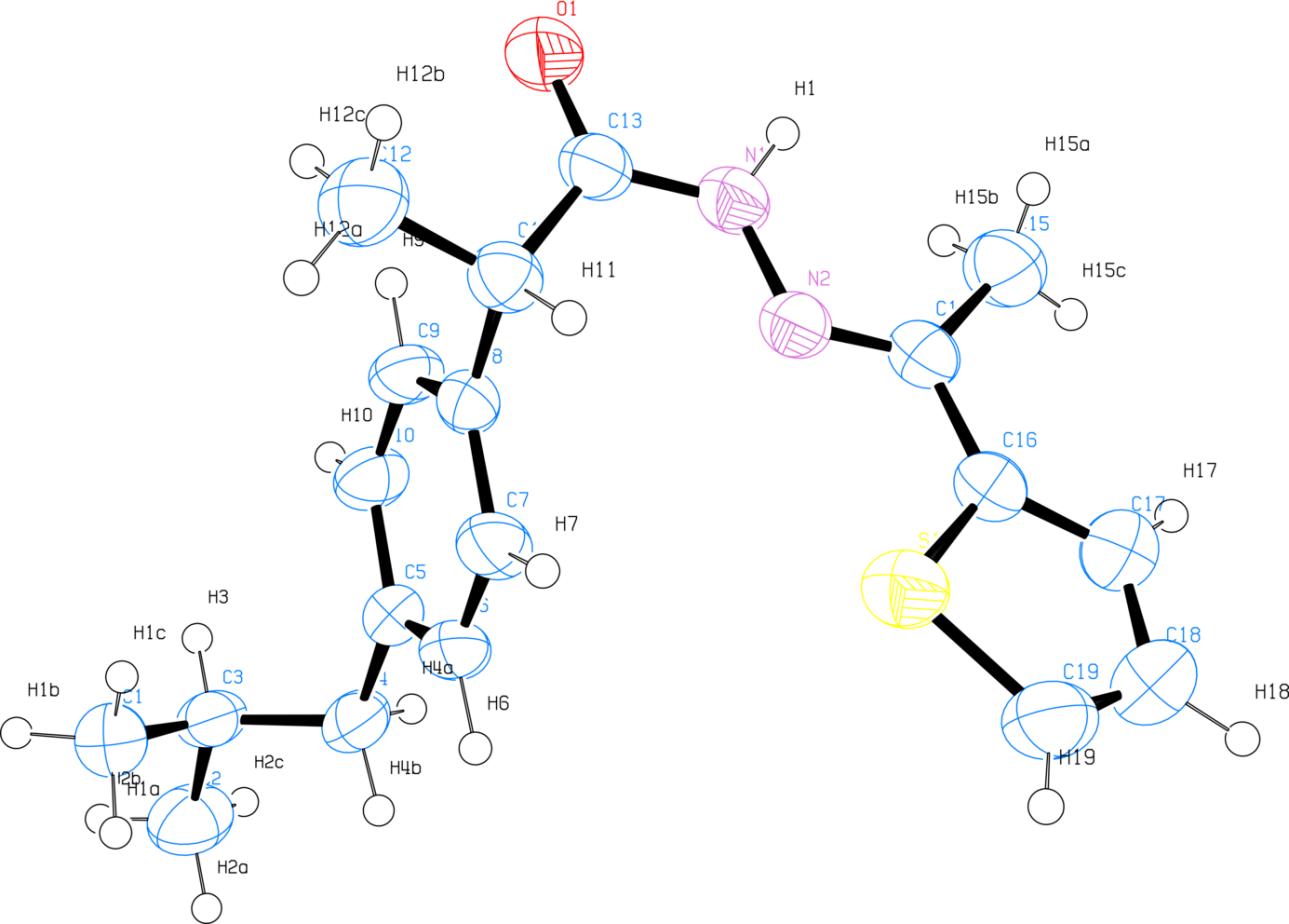
The mol­ecular structure of **1** with displacement ellipsoids drawn at the 50% probability level.

The title compound crystallizes in the triclinic space group *P*ī with one mol­ecule in the asymmetric unit (Fig. 1 ▸) The lengths of the C—S bonds C16—S1 and C19—S1 are 1.7225 (14) and 1.7115 (18) Å, respectively, while the carbonyl bond distance C13—O1 is 1.2297 (15) Å. The torsion angle N1—N2—C14—C16 is 176.95 (10)°, while C8—C11—C13—O1 is 103.88 (14)°. The benzene (*A*, C5–C10; r.m.s.d. = 0.004 Å) and thio­phene (*B*, C16–C19/S1; r.m.s.d. = 0.004 Å) rings are not coplanar, subtending a dihedral angle of 86.69 (4)°.

## 3. Supra­molecular features

**Figure 2:**
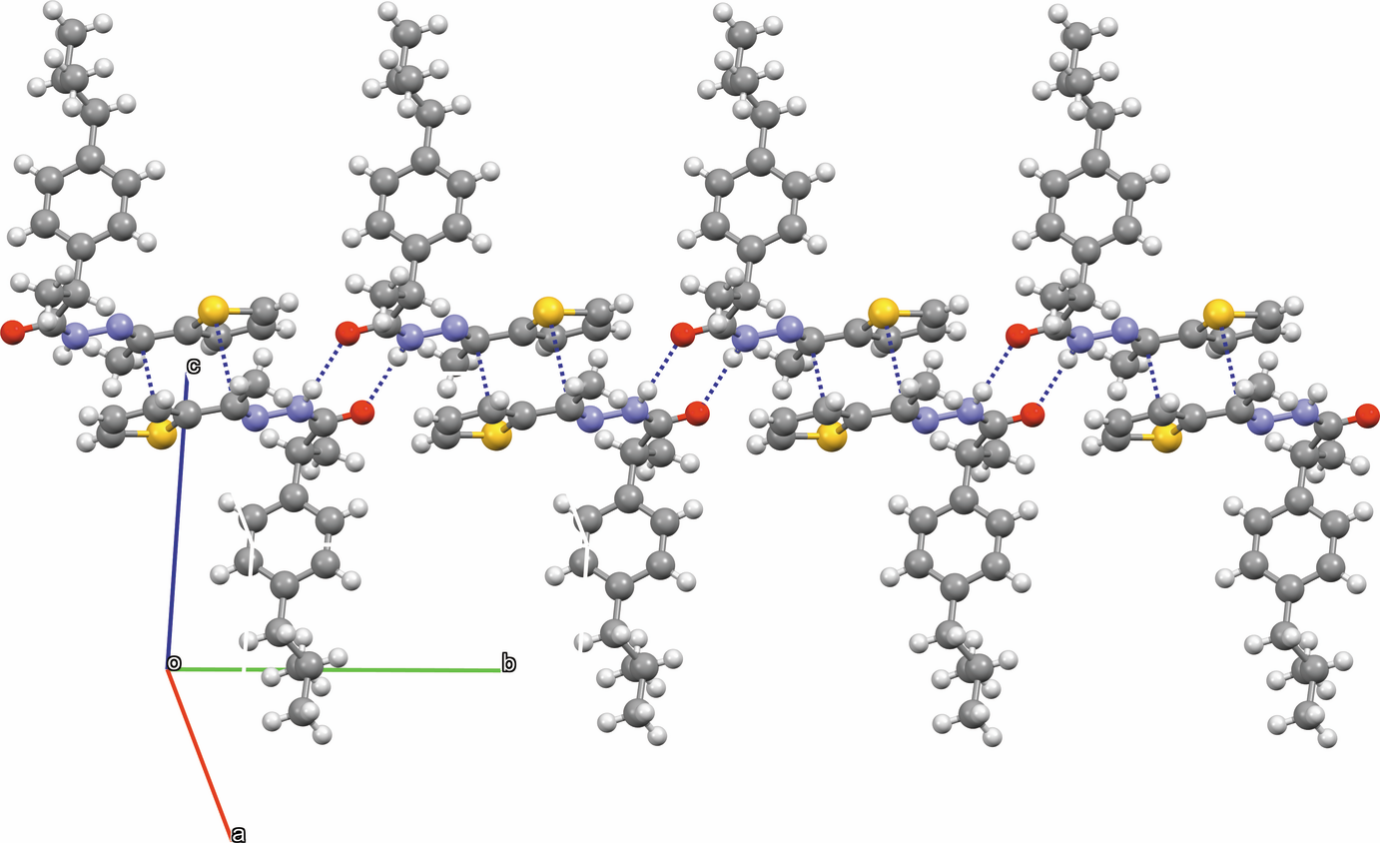
A chain of molecules in the crystal structure of **1**.

In the crystal, N1—H1⋯O1 hydrogen bonds [2.119 (18) Å; Table 1 ▸] lead to the formation of dimers with an 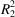
(8) motif. Energy calculations at the B3LYP/6-31G(d, p) level were performed using *Crystal Explorer 21.5* (Mackenzie *et al.*, 2017 ▸; Spackman *et al.*, 2021 ▸) software with the CIF as the input file. The dimer energy was found to be −70.5 kJ mol^−1^. In the crystal, each mol­ecule in the dimer also forms an S1⋯C14 short inter­action [3.5214 (14) Å; symmetry operation −*x*, 2 − y, 2 − *z*], forming another dimer with an 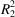
(6) motif and thereby forming a chain running in the *b*-axis direction (Fig. 2 ▸). The energy of the dimer formed by this short inter­action was calculated to be −30.6 kJ mol^−1^.

## 4. Hirshfeld surface analysis

**Figure 3:**
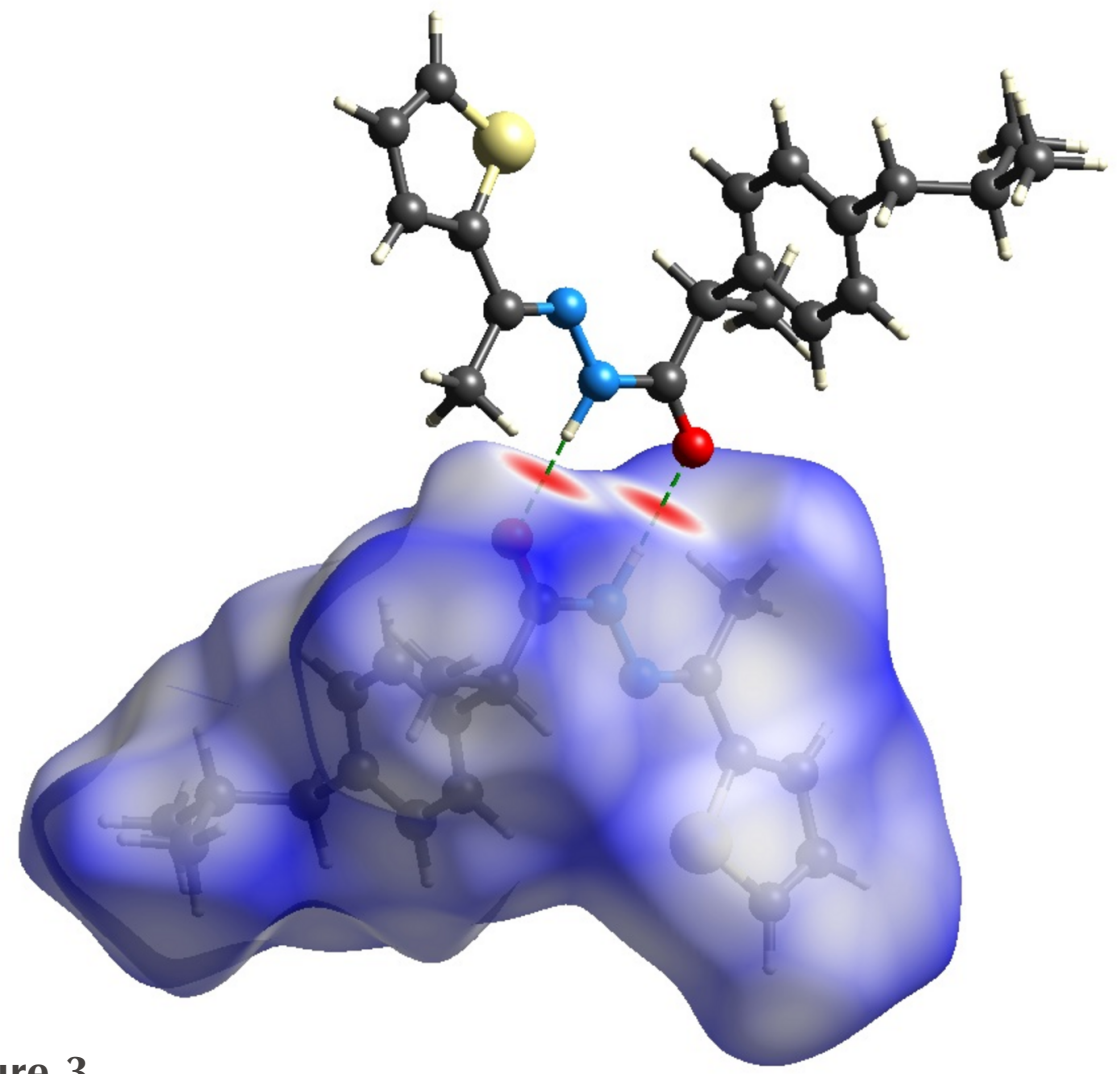
The Hirshfeld surface of the title compound mapped over *d*_norm_ with dashed lines indicating the N—H⋯·O hydrogen bonds that lead to the formation of dimers.

**Figure 4:**
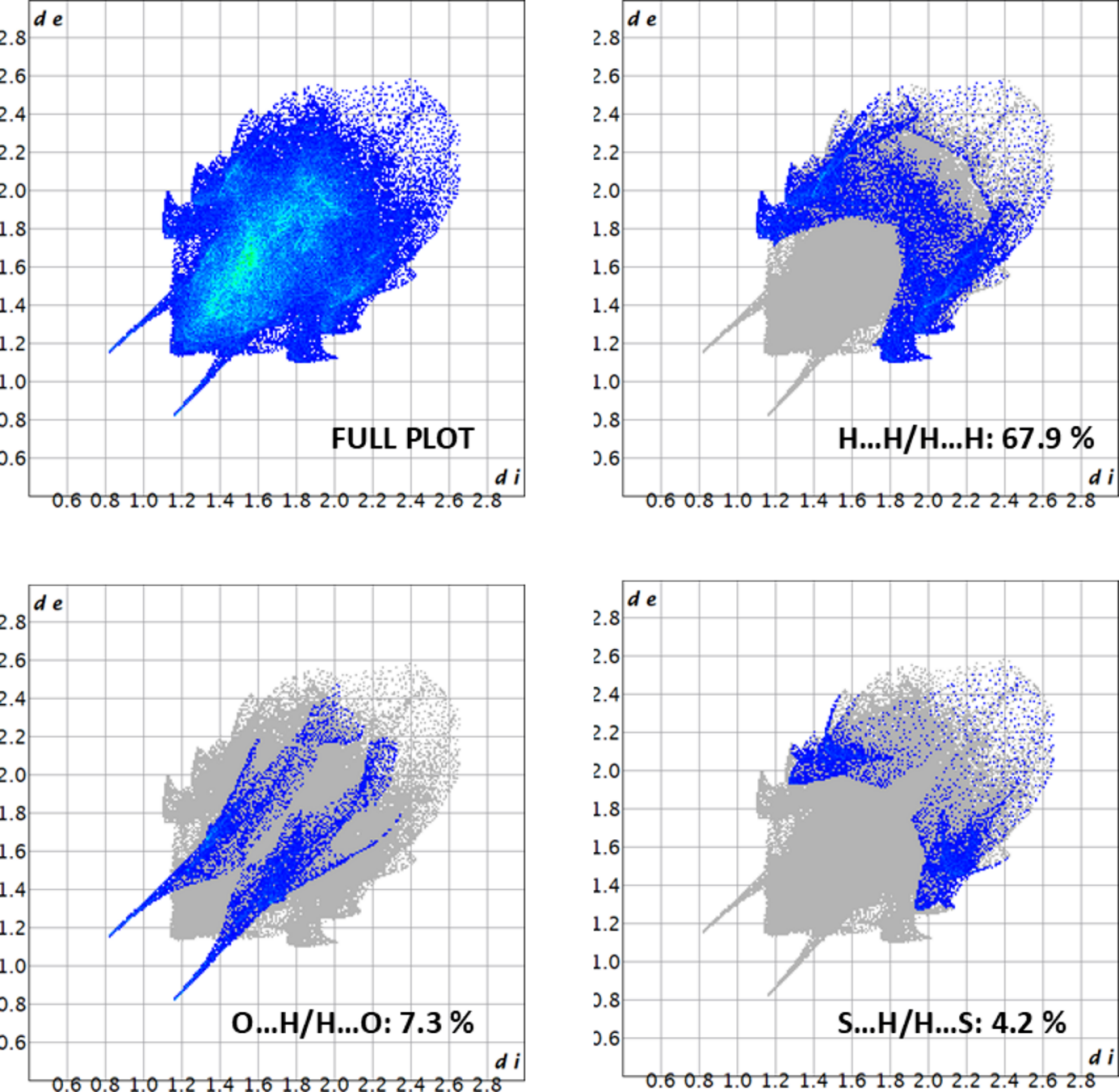
The two-dimensional fingerprint plots of the title mol­ecule, showing all inter­actions and those delineated into H⋯H, O⋯H/H⋯O and S⋯H/ S⋯H.

Hirshfeld surface analysis (Hirshfeld, 1977 ▸; Spackman & Jayatilaka, 2009 ▸) was conducted using *Crystal Explorer* (Spackman *et al.*, 2021 ▸) to visualize and qu­antify the inter­molecular inter­actions in the title mol­ecule. The Hirshfeld surface for the title compound mapped over *d*_norm_ is shown in Fig. 3 ▸. The red region is attributed to the N1—H1⋯O1 inter­action. The two-dimensional fingerprint plots in Fig. 4 ▸ indicate that the major contributions to the crystal packing are from H⋯H (67.9%), C⋯H (13.7%), O⋯H (7.3%) and S⋯H (4.3%) inter­actions.

## 5. Database Survey

A search of the Cambridge Structural Database (CSD, updated to January 2025; Groom *et al.*, 2016 ▸) for the 2-(4-iso­butyl­phen­yl)-*N*′-methyl­propane­hydrazide moiety yielded two closely related structures: 1-[2-(4-iso­butyl­phen­yl)prop­ano­yl]thio­semicarbazide (**2**; CSD refcode HOLQIJ; Fun & Kia *et al.*, 2009 ▸) and *N*-(2,4-dioxo-1,3-thia­zolidin-3-yl)-2-(4-iso­butyl­phen­yl)propenamide (**3**; CSD refcode HUCTUV; Fun & Goh *et al.*, 2009 ▸). In compound **2**, the crystal structure features N—H⋯O and N—H⋯S hydrogen bonds, with donor–acceptor distances of 2.09 (15) and 2.495 (13) Å, respectively, leading to a supra­molecular architecture. In compound **3**, the supra­molecular structure is governed by N—H⋯O hydrogen bonding [1.94 (3) Å], along with several C—H⋯O inter­actions.

## 6. Synthesis and crystallization

The title compound was obtained by refluxing 2-[4-(2-methyl­prop­yl)phen­yl]propane­hydrazide (0.01 mol) and 2-acetyl thio­phene (0.01 mol) in ethanol (20 ml) by adding a catalytic amount of concentrated sulfuric acid for 1 h. The excess solvent was removed under reduced pressure. The solid product obtained was filtered, washed with ethanol, and dried. Single crystals suitable for X-ray analysis were obtained by slow evaporation from an ethanol solution with 80% yield and melting point 340–342 K.

## 7. Refinement

Crystal data, data collection and structure refinement details are summarized in Table 2 ▸. H atoms were refined using a DFIX restraint to ensure chemically reasonable bond lengths and angles, with their *U*_iso_(H) values constrained to 1.5 times the *U*_eq_ of their pivot atoms for terminal *sp*^3^ carbon atoms and 1.2 times for all other carbon atoms.

## Supplementary Material

Crystal structure: contains datablock(s) global, I. DOI: 10.1107/S2056989025003329/dx2065sup1.cif

Structure factors: contains datablock(s) I. DOI: 10.1107/S2056989025003329/dx2065Isup2.hkl

Supporting information file. DOI: 10.1107/S2056989025003329/dx2065Isup3.cml

CCDC reference: 2443395

Additional supporting information:  crystallographic information; 3D view; checkCIF report

## Figures and Tables

**Table 1:** Hydrogen-bond geometry (Å, °)

*D*—H⋯*A*	*D*—H	H⋯*A*	*D*⋯*A*	*D*—H⋯*A*
N1—H1⋯O1^i^	0.859 (18)	2.119 (18)	2.9537 (15)	163.6 (16)

Symmetry code: (i) 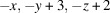.

**Table 2:** Experimental details

Crystal data	
Chemical formula	C_19_H_24_N_2_OS
*M*_r_	328.46
Crystal system, space group	Triclinic, *P* 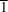
Temperature (K)	293
*a*, *b*, *c* (Å)	7.9057 (3), 10.1929 (3), 12.2794 (3)
α, β, γ (°)	83.238 (1), 89.201 (1), 71.122 (1)
*V* (Å^3^)	929.45 (5)
*Z*	2
Radiation type	Mo *K*α
μ (mm^−1^)	0.18
Crystal size (mm)	0.77 × 0.66 × 0.55

Data collection	
Diffractometer	Bruker D8 Quest Eco
Absorption correction	Multi-scan (*SADABS*, Krause *et al.*, 2015 ▸)
*T*_min_, *T*_max_	0.874, 0.907
No. of measured, independent and observed [*I* > 2σ(*I*)] reflections	27745, 4620, 4098
*R*_int_	0.021
(sin θ/λ)_max_ (Å^−1^)	0.667

Refinement	
*R*[*F*^2^ > 2σ(*F*^2^)], *wR*(*F*^2^), *S*	0.041, 0.123, 1.03
No. of reflections	4620
No. of parameters	304
H-atom treatment	H atoms treated by a mixture of independent and constrained refinement
Δρ_max_, Δρ_min_ (e Å^−3^)	0.25, −0.33

Computer programs: *APEX2* and *SAINT* (Bruker 2012 ▸), *SHELXT* (Sheldrick, 2015*a* ▸), *SHELXL*(Sheldrick, 2015*b* ▸), *ORTEP-3 for Windows* and *WinGX* (Farrugia, 2012 ▸) and *publCIF* (Westrip, 2010 ▸).

## supplementary crystallographic information

### 2-[4-(2-Methylpropyl)phenyl]-N'-[(1Z)-1-(thiophen-2-yl)ethylidene]propanehydrazide Crystal data

**Table:**

C_19_H_24_N_2_OS	*Z* = 2
*M**_r_* = 328.46	*F*(000) = 352
Triclinic, *P*1	*D*_x_ = 1.174 Mg m^−^^3^
Hall symbol: -P 1	Mo *K*α radiation, λ = 0.71073 Å
*a* = 7.9057 (3) Å	Cell parameters from 27745 reflections
*b* = 10.1929 (3) Å	θ = 2.7–28.3°
*c* = 12.2794 (3) Å	µ = 0.18 mm^−^^1^
α = 83.238 (1)°	*T* = 293 K
β = 89.201 (1)°	BLOCK, white
γ = 71.122 (1)°	0.77 × 0.66 × 0.55 mm
*V* = 929.45 (5) Å^3^	

### 2-[4-(2-Methylpropyl)phenyl]-N'-[(1Z)-1-(thiophen-2-yl)ethylidene]propanehydrazide Data collection

**Table:**

Bruker D8 Quest Eco diffractometer	4098 reflections with *I* > 2σ(*I*)
Graphite monochromator	*R*_int_ = 0.021
phi and ω scans	θ_max_ = 28.3°, θ_min_ = 2.7°
Absorption correction: multi-scan (SADABS, Krause *et al.*, 2015)	*h* = −10→10
*T*_min_ = 0.874, *T*_max_ = 0.907	*k* = −13→13
27745 measured reflections	*l* = −16→16
4620 independent reflections	

### 2-[4-(2-Methylpropyl)phenyl]-N'-[(1Z)-1-(thiophen-2-yl)ethylidene]propanehydrazide Refinement

**Table:**

Refinement on *F*^2^	Primary atom site location: structure-invariant direct methods
Least-squares matrix: full	Secondary atom site location: difference Fourier map
*R*[*F*^2^ > 2σ(*F*^2^)] = 0.041	Hydrogen site location: inferred from neighbouring sites
*wR*(*F*^2^) = 0.123	H atoms treated by a mixture of independent and constrained refinement
*S* = 1.03	*w* = 1/[σ^2^(*F*_o_^2^) + (0.0654*P*)^2^ + 0.1712*P*] where *P* = (*F*_o_^2^ + 2*F*_c_^2^)/3
4620 reflections	(Δ/σ)_max_ = 0.001
304 parameters	Δρ_max_ = 0.25 e Å^−^^3^
0 restraints	Δρ_min_ = −0.33 e Å^−^^3^
0 constraints	

### 2-[4-(2-Methylpropyl)phenyl]-N'-[(1Z)-1-(thiophen-2-yl)ethylidene]propanehydrazide Special details

**Table:**

Geometry. All esds (except the esd in the dihedral angle between two l.s. planes) are estimated using the full covariance matrix. The cell esds are taken into account individually in the estimation of esds in distances, angles and torsion angles; correlations between esds in cell parameters are only used when they are defined by crystal symmetry. An approximate (isotropic) treatment of cell esds is used for estimating esds involving l.s. planes.
Refinement. Refinement of F^2^ against ALL reflections. The weighted R-factor wR and goodness of fit S are based on F^2^, conventional R-factors R are based on F, with F set to zero for negative F^2^. The threshold expression of F^2^ > 2sigma(F^2^) is used only for calculating R-factors(gt) etc. and is not relevant to the choice of reflections for refinement. R-factors based on F^2^ are statistically about twice as large as those based on F, and R- factors based on ALL data will be even larger.

### 2-[4-(2-Methylpropyl)phenyl]-N'-[(1Z)-1-(thiophen-2-yl)ethylidene]propanehydrazide Fractional atomic coordinates and isotropic or equivalent isotropic displacement parameters (Å^2^)

**Table:**

	*x*	*y*	*z*	*U*_iso_*/*U*_eq_	
H7	0.214 (2)	1.0985 (18)	0.6861 (13)	0.061 (4)*	
H11	−0.014 (2)	1.2611 (16)	0.7709 (12)	0.051 (4)*	
H3	0.7134 (19)	1.3440 (16)	0.4536 (12)	0.050 (4)*	
H10	0.538 (2)	1.4079 (17)	0.6116 (13)	0.061 (4)*	
H4B	0.744 (2)	1.0670 (19)	0.5259 (14)	0.064 (4)*	
H12B	−0.225 (3)	1.480 (2)	0.7002 (18)	0.093 (6)*	
H6	0.487 (2)	1.0259 (18)	0.6006 (14)	0.064 (4)*	
H9	0.267 (2)	1.4757 (18)	0.6996 (14)	0.066 (5)*	
H4A	0.816 (2)	1.1609 (18)	0.5930 (15)	0.066 (5)*	
H1A	0.603 (2)	1.173 (2)	0.3246 (15)	0.073 (5)*	
H1C	0.468 (3)	1.291 (2)	0.3788 (16)	0.081 (6)*	
H2B	0.929 (3)	1.263 (2)	0.3186 (18)	0.085 (6)*	
H2C	1.001 (3)	1.205 (2)	0.438 (2)	0.102 (8)*	
H12A	−0.118 (3)	1.413 (2)	0.6042 (19)	0.090 (6)*	
H1B	0.591 (3)	1.327 (2)	0.2852 (18)	0.085 (6)*	
H1	0.107 (2)	1.3514 (19)	1.0060 (15)	0.068 (5)*	
H19	0.276 (3)	0.649 (2)	0.9227 (19)	0.099 (7)*	
H12C	−0.080 (3)	1.536 (2)	0.6532 (18)	0.093 (7)*	
H2A	0.940 (3)	1.108 (2)	0.3691 (17)	0.089 (6)*	
H17	0.426 (3)	0.840 (2)	1.1552 (19)	0.087 (6)*	
H15A	0.198 (4)	1.177 (3)	1.166 (2)	0.134 (10)*	
H15B	0.342 (4)	1.212 (3)	1.113 (3)	0.140 (10)*	
H15C	0.371 (4)	1.072 (3)	1.171 (2)	0.121 (9)*	
H18	0.433 (3)	0.615 (2)	1.1025 (19)	0.100 (7)*	
S1	0.20178 (5)	0.88866 (4)	0.90928 (3)	0.05992 (13)	
N2	0.16268 (14)	1.17435 (11)	0.94142 (8)	0.0464 (2)	
C8	0.20740 (16)	1.29536 (11)	0.69966 (9)	0.0410 (2)	
N1	0.11506 (16)	1.31606 (11)	0.94519 (9)	0.0499 (2)	
C6	0.4414 (2)	1.11989 (12)	0.61919 (10)	0.0515 (3)	
O1	−0.01124 (18)	1.53227 (10)	0.86090 (8)	0.0697 (3)	
C11	0.02513 (17)	1.34485 (13)	0.75071 (10)	0.0464 (3)	
C9	0.30698 (17)	1.38476 (12)	0.67826 (11)	0.0481 (3)	
C10	0.46957 (18)	1.34333 (12)	0.62781 (11)	0.0509 (3)	
C5	0.54019 (17)	1.20942 (11)	0.59659 (9)	0.0452 (3)	
C7	0.2783 (2)	1.16138 (13)	0.67006 (10)	0.0508 (3)	
C16	0.27929 (17)	0.94374 (13)	1.02006 (10)	0.0493 (3)	
C4	0.71791 (18)	1.16349 (13)	0.54132 (12)	0.0532 (3)	
C14	0.24044 (16)	1.09260 (13)	1.02689 (10)	0.0471 (3)	
C13	0.03963 (17)	1.40564 (13)	0.85616 (10)	0.0483 (3)	
C3	0.73007 (17)	1.25536 (12)	0.43557 (11)	0.0504 (3)	
C19	0.2924 (3)	0.71716 (17)	0.96246 (16)	0.0715 (4)	
C15	0.2901 (3)	1.1379 (2)	1.13036 (14)	0.0667 (4)	
C17	0.3711 (2)	0.83168 (17)	1.09363 (14)	0.0662 (4)	
C1	0.5877 (2)	1.26297 (17)	0.35180 (13)	0.0626 (4)	
C12	−0.1107 (2)	1.4535 (2)	0.67142 (15)	0.0662 (4)	
C2	0.9153 (2)	1.20371 (19)	0.38798 (19)	0.0730 (5)	
C18	0.3772 (3)	0.70272 (18)	1.05916 (17)	0.0793 (5)	

### 2-[4-(2-Methylpropyl)phenyl]-N'-[(1Z)-1-(thiophen-2-yl)ethylidene]propanehydrazide Atomic displacement parameters (Å^2^)

**Table:**

	*U*^11^	*U*^22^	*U*^33^	*U*^12^	*U*^13^	*U*^23^
S1	0.0657 (2)	0.0562 (2)	0.0589 (2)	−0.01866 (16)	0.00501 (16)	−0.01461 (15)
N2	0.0515 (5)	0.0443 (5)	0.0446 (5)	−0.0157 (4)	0.0091 (4)	−0.0100 (4)
C8	0.0512 (6)	0.0374 (5)	0.0349 (5)	−0.0146 (4)	0.0009 (4)	−0.0062 (4)
N1	0.0637 (6)	0.0459 (5)	0.0423 (5)	−0.0181 (5)	0.0081 (4)	−0.0131 (4)
C6	0.0726 (8)	0.0343 (5)	0.0478 (6)	−0.0158 (5)	0.0117 (6)	−0.0113 (4)
O1	0.1087 (9)	0.0434 (5)	0.0536 (5)	−0.0177 (5)	0.0126 (5)	−0.0136 (4)
C11	0.0506 (6)	0.0452 (6)	0.0464 (6)	−0.0183 (5)	0.0035 (5)	−0.0101 (5)
C9	0.0554 (7)	0.0323 (5)	0.0572 (7)	−0.0135 (5)	0.0081 (5)	−0.0107 (5)
C10	0.0545 (7)	0.0363 (5)	0.0632 (7)	−0.0163 (5)	0.0087 (6)	−0.0077 (5)
C5	0.0543 (6)	0.0348 (5)	0.0413 (5)	−0.0081 (4)	0.0028 (5)	−0.0019 (4)
C7	0.0706 (8)	0.0403 (6)	0.0494 (6)	−0.0263 (6)	0.0123 (6)	−0.0134 (5)
C16	0.0489 (6)	0.0508 (6)	0.0459 (6)	−0.0128 (5)	0.0103 (5)	−0.0078 (5)
C4	0.0526 (7)	0.0384 (6)	0.0581 (7)	−0.0022 (5)	0.0054 (5)	−0.0006 (5)
C14	0.0454 (6)	0.0516 (6)	0.0437 (6)	−0.0141 (5)	0.0089 (4)	−0.0098 (5)
C13	0.0564 (7)	0.0447 (6)	0.0460 (6)	−0.0176 (5)	0.0111 (5)	−0.0116 (5)
C3	0.0542 (7)	0.0345 (5)	0.0573 (7)	−0.0073 (5)	0.0124 (5)	−0.0062 (5)
C19	0.0836 (11)	0.0522 (8)	0.0815 (11)	−0.0230 (7)	0.0200 (9)	−0.0180 (7)
C15	0.0766 (10)	0.0660 (9)	0.0523 (8)	−0.0127 (8)	−0.0068 (7)	−0.0152 (7)
C17	0.0760 (10)	0.0569 (8)	0.0569 (8)	−0.0110 (7)	0.0005 (7)	−0.0022 (6)
C1	0.0711 (9)	0.0559 (8)	0.0540 (8)	−0.0124 (7)	0.0055 (7)	−0.0043 (6)
C12	0.0561 (8)	0.0679 (9)	0.0691 (9)	−0.0121 (7)	−0.0106 (7)	−0.0087 (8)
C2	0.0617 (9)	0.0606 (9)	0.0887 (12)	−0.0100 (7)	0.0251 (9)	−0.0085 (8)
C18	0.0958 (13)	0.0514 (8)	0.0786 (11)	−0.0105 (8)	0.0100 (9)	0.0008 (8)

### 2-[4-(2-Methylpropyl)phenyl]-N'-[(1Z)-1-(thiophen-2-yl)ethylidene]propanehydrazide Geometric parameters (Å, º)

**Table:**

S1—C19	1.7115 (18)	C4—C3	1.5300 (18)
S1—C16	1.7225 (14)	C4—H4B	0.978 (18)
N2—C14	1.2862 (16)	C4—H4A	1.003 (18)
N2—N1	1.3761 (14)	C14—C15	1.5005 (19)
C8—C9	1.3860 (17)	C3—C1	1.512 (2)
C8—C7	1.3881 (15)	C3—C2	1.521 (2)
C8—C11	1.5175 (17)	C3—H3	0.923 (15)
N1—C13	1.3473 (17)	C19—C18	1.341 (3)
N1—H1	0.859 (19)	C19—H19	0.93 (2)
C6—C5	1.3841 (18)	C15—H15A	0.85 (3)
C6—C7	1.3853 (19)	C15—H15B	0.97 (4)
C6—H6	0.962 (17)	C15—H15C	0.87 (3)
O1—C13	1.2297 (15)	C17—C18	1.414 (3)
C11—C13	1.5208 (17)	C17—H17	0.90 (2)
C11—C12	1.527 (2)	C1—H1A	0.98 (2)
C11—H11	1.002 (16)	C1—H1C	0.96 (2)
C9—C10	1.3793 (18)	C1—H1B	0.99 (2)
C9—H9	0.945 (17)	C12—H12B	0.93 (2)
C10—C5	1.3949 (16)	C12—H12A	0.98 (2)
C10—H10	0.979 (17)	C12—H12C	0.95 (2)
C5—C4	1.5087 (18)	C2—H2B	1.01 (2)
C7—H7	0.942 (18)	C2—H2C	0.92 (3)
C16—C17	1.373 (2)	C2—H2A	0.99 (2)
C16—C14	1.4599 (18)	C18—H18	0.96 (2)
			
C19—S1—C16	91.71 (8)	O1—C13—C11	121.97 (12)
C14—N2—N1	117.92 (10)	N1—C13—C11	117.84 (11)
C9—C8—C7	117.67 (11)	C1—C3—C2	110.69 (14)
C9—C8—C11	120.64 (10)	C1—C3—C4	111.69 (12)
C7—C8—C11	121.67 (11)	C2—C3—C4	110.71 (12)
C13—N1—N2	120.01 (10)	C1—C3—H3	108.8 (9)
C13—N1—H1	116.0 (12)	C2—C3—H3	107.3 (9)
N2—N1—H1	122.2 (12)	C4—C3—H3	107.4 (9)
C5—C6—C7	121.56 (11)	C18—C19—S1	112.06 (14)
C5—C6—H6	119.9 (10)	C18—C19—H19	130.0 (14)
C7—C6—H6	118.5 (10)	S1—C19—H19	118.0 (14)
C8—C11—C13	109.93 (10)	C14—C15—H15A	112 (2)
C8—C11—C12	111.19 (12)	C14—C15—H15B	110.5 (19)
C13—C11—C12	110.02 (11)	H15A—C15—H15B	102 (3)
C8—C11—H11	107.7 (8)	C14—C15—H15C	113.9 (18)
C13—C11—H11	107.3 (9)	H15A—C15—H15C	113 (3)
C12—C11—H11	110.6 (9)	H15B—C15—H15C	105 (2)
C10—C9—C8	121.41 (10)	C16—C17—C18	112.44 (16)
C10—C9—H9	117.7 (11)	C16—C17—H17	123.3 (14)
C8—C9—H9	120.9 (11)	C18—C17—H17	124.2 (14)
C9—C10—C5	121.13 (11)	C3—C1—H1A	113.0 (11)
C9—C10—H10	120.7 (9)	C3—C1—H1C	114.2 (12)
C5—C10—H10	118.1 (10)	H1A—C1—H1C	104.8 (16)
C6—C5—C10	117.32 (11)	C3—C1—H1B	111.6 (12)
C6—C5—C4	121.29 (11)	H1A—C1—H1B	104.6 (16)
C10—C5—C4	121.38 (11)	H1C—C1—H1B	108.0 (16)
C6—C7—C8	120.91 (11)	C11—C12—H12B	111.8 (14)
C6—C7—H7	120.5 (10)	C11—C12—H12A	108.8 (13)
C8—C7—H7	118.6 (10)	H12B—C12—H12A	107.1 (18)
C17—C16—C14	129.06 (13)	C11—C12—H12C	112.5 (14)
C17—C16—S1	110.69 (11)	H12B—C12—H12C	107.5 (18)
C14—C16—S1	120.23 (10)	H12A—C12—H12C	109.0 (19)
C5—C4—C3	114.56 (10)	C3—C2—H2B	112.8 (11)
C5—C4—H4B	109.1 (10)	C3—C2—H2C	110.4 (15)
C3—C4—H4B	109.4 (10)	H2B—C2—H2C	106.9 (19)
C5—C4—H4A	110.0 (10)	C3—C2—H2A	109.9 (12)
C3—C4—H4A	108.2 (10)	H2B—C2—H2A	107.1 (17)
H4B—C4—H4A	105.1 (13)	H2C—C2—H2A	109.6 (19)
N2—C14—C16	115.38 (11)	C19—C18—C17	113.10 (16)
N2—C14—C15	125.65 (13)	C19—C18—H18	124.1 (14)
C16—C14—C15	118.95 (12)	C17—C18—H18	122.7 (14)
O1—C13—N1	120.17 (12)		
			
C14—N2—N1—C13	177.41 (11)	N1—N2—C14—C16	176.95 (10)
C9—C8—C11—C13	−53.84 (15)	N1—N2—C14—C15	−2.2 (2)
C7—C8—C11—C13	127.93 (12)	C17—C16—C14—N2	175.00 (14)
C9—C8—C11—C12	68.24 (15)	S1—C16—C14—N2	−6.57 (15)
C7—C8—C11—C12	−109.99 (14)	C17—C16—C14—C15	−5.8 (2)
C7—C8—C9—C10	0.99 (19)	S1—C16—C14—C15	172.59 (12)
C11—C8—C9—C10	−177.31 (12)	N2—N1—C13—O1	176.36 (12)
C8—C9—C10—C5	−0.2 (2)	N2—N1—C13—C11	−5.30 (17)
C7—C6—C5—C10	0.43 (19)	C8—C11—C13—O1	103.88 (14)
C7—C6—C5—C4	179.90 (12)	C12—C11—C13—O1	−18.90 (18)
C9—C10—C5—C6	−0.56 (19)	C8—C11—C13—N1	−74.43 (14)
C9—C10—C5—C4	179.97 (12)	C12—C11—C13—N1	162.80 (13)
C5—C6—C7—C8	0.4 (2)	C5—C4—C3—C1	−57.96 (16)
C9—C8—C7—C6	−1.11 (19)	C5—C4—C3—C2	178.20 (14)
C11—C8—C7—C6	177.17 (12)	C16—S1—C19—C18	−0.08 (15)
C19—S1—C16—C17	0.06 (12)	C14—C16—C17—C18	178.51 (14)
C19—S1—C16—C14	−178.63 (11)	S1—C16—C17—C18	−0.04 (19)
C6—C5—C4—C3	123.70 (14)	S1—C19—C18—C17	0.1 (2)
C10—C5—C4—C3	−56.85 (18)	C16—C17—C18—C19	0.0 (2)

### 2-[4-(2-Methylpropyl)phenyl]-N'-[(1Z)-1-(thiophen-2-yl)ethylidene]propanehydrazide Hydrogen-bond geometry (Å, º)

**Table:**

*D*—H···*A*	*D*—H	H···*A*	*D*···*A*	*D*—H···*A*
N1—H1···O1^i^	0.859 (18)	2.119 (18)	2.9537 (15)	163.6 (16)

Symmetry code: (i) −*x*, −*y*+3, −*z*+2.

### Table 2. The energy values (eV) of global reactivity descriptors for the title compound.

**Table:**

Global reactivity Descriptors	Calculated values (eV)
E(HOMO)	-6.0826
E(LUMO)	-1.7796
Energy gap	4.303
Electron affinity (A)	1.7796
Ionisation energy (I)	6.0826
Electronegativity (χ)	3.9311
Chemical hardness (η)	2.1515
Chemical potential (µ)	-3.9311
Chemical hardness (S)	0.2324
Electrophilicity index (ω)	3.5913
